# Transcription Factor Binding Site Polymorphism in the *Motilin* Gene Associated with Left-Sided Displacement of the Abomasum in German Holstein Cattle

**DOI:** 10.1371/journal.pone.0035562

**Published:** 2012-04-20

**Authors:** Stefanie Mömke, Marlene Sickinger, Jürgen Rehage, Klaus Doll, Ottmar Distl

**Affiliations:** 1 Institute for Animal Breeding and Genetics, University of Veterinary Medicine Hannover, Hannover, Germany; 2 Clinic for Ruminants and Swine, Faculty for Veterinary Medicine, Justus-Liebig-University Giessen, Giessen, Germany; 3 Clinic for Cattle, University for Veterinary Medicine Hannover, Hannover, Germany; University of Queensland, Australia

## Abstract

Left-sided displacement of the abomasum (LDA) is a common disease in many dairy cattle breeds. A genome-wide screen for QTL for LDA in German Holstein (GH) cows indicated *motilin* (*MLN*) as a candidate gene on bovine chromosome 23. Genomic DNA sequence analysis of *MLN* revealed a total of 32 polymorphisms. All informative polymorphisms used for association analyses in a random sample of 1,136 GH cows confirmed *MLN* as a candidate for LDA. A single nucleotide polymorphism (FN298674:g.90T>C) located within the first non-coding exon of bovine *MLN* affects a NKX2-5 transcription factor binding site and showed significant associations (OR_allele_ = 0.64; −log_10_P_allele_ = 6.8, −log_10_P_genotype_ = 7.0) with LDA. An expression study gave evidence of a significantly decreased *MLN* expression in cows carrying the mutant allele (C). In individuals heterozygous or homozygous for the mutation, *MLN* expression was decreased by 89% relative to the wildtype. FN298674:g.90T>C may therefore play a role in bovine LDA via the motility of the abomasum. This *MLN* SNP appears useful to reduce the incidence of LDA in German Holstein cattle and provides a first step towards a deeper understanding of the genetics of LDA.

## Introduction

Left-sided displacement of the abomasum (LDA) is a common dairy cattle disease with prevalences of about 2% in the German Holstein (GH) population [1], [2]. In the course of LDA the abomasum starts bloating and displaces from the dexter abdominal floor to the sinistral abdominal wall. LDA has to be treated surgically by fixing the abomasum into its correct position. However, even if the treatment succeeded without any incidents, a significantly reduced milk performance as well as conception problems increase the culling risk [1]–[3]. On the average, about one half of all cows affected by LDA were culled within the first year after surgery [1]. Thus, LDA increases veterinary costs for dairy farms and decreases life expectancy for dairy cows. Several QTL for LDA have already been identified in GH cows [4], among them a QTL proximal on bovine chromosome (BTA) 23. Since LDA is usually preceded by a decreased motility of the abomasum, impaired abomasal emptying, and malfunctions at the level of the intrinsic nervous system combined with impaired cholinergic muscle responses [3], the *motilin (MLN)* gene located proximally on BTA23 was an appropriate candidate gene. *MLN* encodes a small peptide hormone regulating interdigestive gastrointestinal contraction [5]. In human, *MLN* is mainly expressed in the stomach (UniGene, EST profile, HS.2813), which is the analog to the bovine abomasum. A reduced expression of *MLN* might lead to an insufficient gastrointestinal motility. This is also supported by results in human pharmacology, where medications based on *MLN*-agonists can remedy conditions like the diabetic gastroparesis [6], [7]. Also in cows undergoing surgical correction of LDA, the motilin agonist erythromycin increases the abomasal emptying rate by binding to motilin receptors [8].

LDA is a multifactorial disease, in which environmental effects play a role such as twin births [1], [9], housing system [1], and feeding factors [10]. However, the heritability of LDA reported in various studies ranged between 20% and 50% [1], [2] and thus is much higher than in any other common cattle disease.

The objective of this study was to investigate *MLN* as the most promising candidate gene for LDA on proximal BTA23. We were able to detect a significantly LDA-associated single nucleotide polymorphism (SNP) affecting a predicted transcription factor binding site of the *MLN* gene which also changes the expression level of this gene. Therefore, this polymorphism is useful for a genetic test to reduce the incidence of LDA in GH cows.

## Results

### Mutation analysis

**Figure 1:**
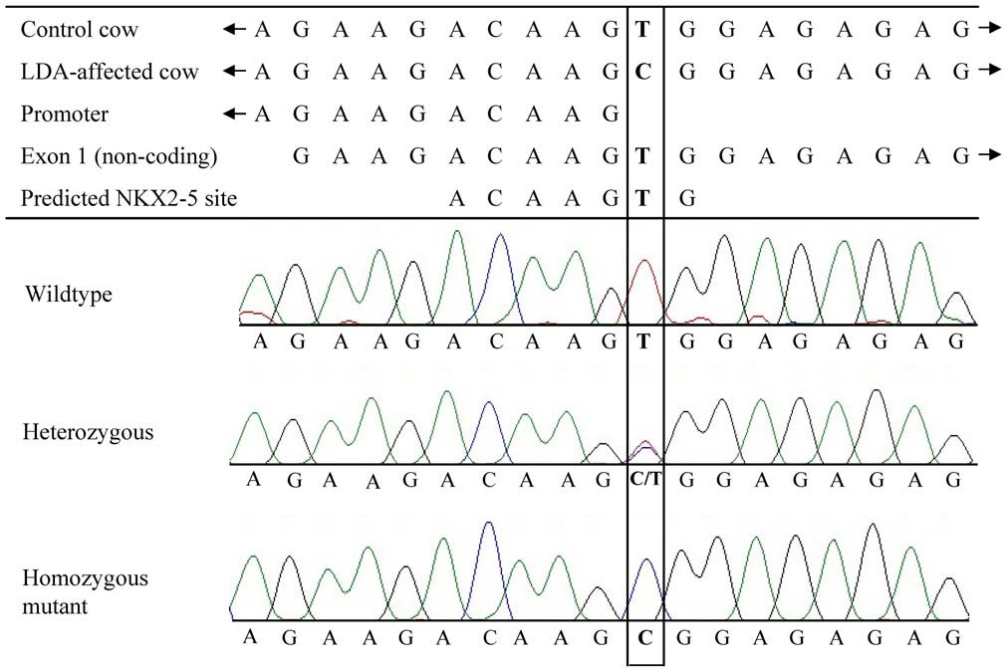
Mutation within the bovine *motilin (MLN)* gene associated with LDA. Sequence of *MLN* surrounding the FN298674:g.90T>C SNP for the wildtype (control cow) and the mutated allele (LDA-affected cow) as well as the 3′ end of the predicted *MLN* promoter, the 5′ start sequence of the non-coding first exon of *MLN* and the NKX2-5 transcription factor binding site are shown. Furthermore, chromatograms of this section are given for each one of the homozygous wildtype, heterozygous and homozygous mutant individuals.

First, comparative sequencing of the complete *MLN* gene including all exons and introns as well as the 3′ and 5′ UTR sequences was done in four GH cows affected by LDA and four control cows of the same breed. The genomic sequence of the control cows (FN298674) was not in complete agreement with the corresponding sequence of the *Bos taurus* genome 4.0 (NC_007324). In comparison to the reference sequence, the first intron of *MLN* was shorter by 557 bp in all GH cows sequenced. Eight German Fleckvieh cattle were also analysed for this sequence variation of *MLN* with the result of an identical length of intron 1 as the GH cows. The putative promoter of bovine *MLN* extends from 73 bp to 123 bp using the genomic *MLN* sequence identified here (FN298674). We detected a total of 32 polymorphisms, of which 30 were SNPs and two were short tandem repeats. All polymorphisms were tested for their information content in a sample of 48 GH cows, with one half of them affected by LDA. Only eleven polymorphisms were informative (Table 1). None of the identified SNPs is located within the coding sequence of *MLN*, but FN298674:g.62G>A is located within the promoter and FN298674:g.90T>C within the non-coding first exon of *MLN* and the latter one affects a predicted NKX2-5 transcription factor binding site (Figure 1).

**Table 1:** All identified polymorphisms with their location within *motilin* (*MLN*).

SNP/microsatellite	I	Location of polymorphism in *MLN*	Primer F (5′>3′)	Primer R (5′>3′)	AT	PS
FN298674:g.62G>A	+	promoter	CAGGAATCCAGACTCCTCAC	CCGGCTCTTTTGTTAACTT	60	521
FN298674:g.90T>C	+	exon 1	CAGGAATCCAGACTCCTCAC	CCGGCTCTTTTGTTAACTT	60	521
FN298674:g.551(AG)45	+	intron 1	GCTCCGGGAAGTTAACAAAA	GAGCTGCTGACTTTGCCTTT *	59	241
FN298674:g.658A>G		intron 1	GCTCCGGGAAGTTAACAAAA	ACATTTAATTCACCCATAAGAATCAA	59	403
FN298674:g.684G>A		intron 1	GCTCCGGGAAGTTAACAAAA	ACATTTAATTCACCCATAAGAATCAA	59	403
FN298674:g.707G>A		intron 1	GCTCCGGGAAGTTAACAAAA	ACATTTAATTCACCCATAAGAATCAA	59	403
FN298674:g.869T>A		intron 1	GAAGTTAACAAAAGAGCCGG	ACCCTCCCTTTGGAGAGAAT	60	822
FN298674:g.1052C>T		intron 1	GAAGTTAACAAAAGAGCCGG	ACCCTCCCTTTGGAGAGAAT	60	822
FN298674:g.1719A>T		intron 1	GCAGCCTTGGAAGATTCTCT	CATCCCATAAGTGGGTGTGT	60	591
FN298674:g.1891insG	+	intron 1	GACACACCCACTTATGGGAT	TCCCAGCTCCAGCAAGTCTA *	60	155
FN298674:g.2037T>C		intron 1	GACACACCCACTTATGGGAT	TTGTTGGCCCTTCCCTTTG	58	514
FN298674:g.2045C>G	+	intron 1	GACACACCCACTTATGGGAT	TTGTTGGCCCTTCCCTTTG	58	514
FN298674:g.2451T>A		intron 1	GCTCCTGCTTCATTAGCTC	GTGTGTCTGACCTCTCACC	58	779
FN298674:g.2525G>T		intron 1	GCTCCTGCTTCATTAGCTC	GTGTGTCTGACCTCTCACC	58	779
FN298674:g.2929G>A	+	intron 2	GCTCCTGCTTCATTAGCTC	GTGTGTCTGACCTCTCACC	58	779
FN298674:g.2985C>T	+	intron 2	GCTCCTGCTTCATTAGCTC	GTGTGTCTGACCTCTCACC	58	779
FN298674:g.3537(T)23		intron 2	CCCTGGGCCTTTTACCTTAG *	TTGCCATTTCCTTTTCCAAC	60	130
FN298674:g.4189C>T		intron 2	GAGACTGAGGCTCCAACAGG	CTGGCAATTACGAGGAGGAA	60	503
FN298674:g.4360G>C	+	intron 2	GAGACTGAGGCTCCAACAGG	CTGGCAATTACGAGGAGGAA	60	503
FN298674:g.4942insT	+	intron 2	AATCCGAGGACGATGTGAAG *	GCCCTTTGGGGTTTCTATTC	60	128
FN298674:g.5224G>T		intron 3	GCAGCGAGAGGGTGCTTG	AGCTCGTCACCTGAGTGTC	58	795
FN298674:g.5596T>G		intron 3	CACAGAATGTGCTGCGAACT	AATGGATCTCATGGCTCTGG	60	460
FN298674:g.6197G>A		intron 3	GCCCTTCTCCCTCAGCTACT	CCAATTTCCACAGGAGCAGT	60	479
FN298674:g.6689C>T	+	intron 4	ACGCTGCCTTATTTCACAGC	ATGGATCTCTGCAGGAGGAG	60	496
FN298674:g.6728G>A	+	intron 4	CTGGCAGGTTTCAGGCTCT	ATGGATCTCTGCAGGAGGAG	58	672
FN298674:g.6920C>T		intron 4	CTGGCAGGTTTCAGGCTCT	ATGGATCTCTGCAGGAGGAG	58	672
FN298674:g.6984C>T		intron 4	CTGGCAGGTTTCAGGCTCT	ATGGATCTCTGCAGGAGGAG	58	672
FN298674:g.7002T>A		intron 4	TCCGAGCGGAGTCTAATCAC	CTGCCTCCAGCTTTCTGTGT	60	533
FN298674:g.7153C>T		intron 4	TCCGAGCGGAGTCTAATCAC	CTGCCTCCAGCTTTCTGTGT	60	533
FN298674:g.7403delGAC		intron 4	CAGCCTGAGTCACTGGTAC	AGCTCAGAAGGGTCAGCAGATC *	60	245
FN298674:g.7408delA		intron 4	CAGCCTGAGTCACTGGTAC	AGCTCAGAAGGGTCAGCAGATC *	60	245
FN298674:g.7410TCA>CTG		intron 4	CAGCCTGAGTCACTGGTAC	ATTTATGGCCAAACCCTCTC	58	528

* IRD labeled.

Informative (I) polymorphisms are indicated by a plus sign. The primer pairs used are given with their annealing temperatures (AT) and product sizes (PS).

### Association analyses

**Figure 2:**
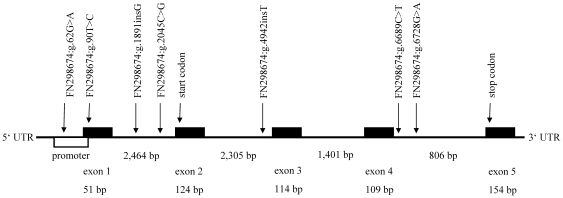
Structure of the motilin (*MLN*) gene and positions of seven SNPs associated with LDA. The figure shows the five exons and the promoter region of the bovine *MLN*. Arrows indicate the positions of the seven polymorphisms associated with LDA in German Holstein cows. The size of the exons and introns is given in base pairs. The positions of the start and stop codons are indicated.

All informative polymorphisms were tested for association with LDA using 601 affected and 535 unaffected GH dairy cows. Seven SNPs (FN298674:g.62G>A, FN298674:g.90T>C, FN298674:g.1891insG, FN298674:g.2045C>G, FN298674:g.4942insT, FN298674:g.6689C>T, FN298674:g.6728G>A; Figure 2) showed significant allelic and genotypic associations with LDA (Table 2). FN298674:g.6689C>T and FN298674:g.6728G>A were in strong linkage disequilibrium (LD) of r^2^ = 1.0 and FN298674:g.90T>C and FN298674:g.1891insG showed an LD of r^2^ = 0.8 (Figure S1). The odds ratio (OR) was highest for FN298674:g.1891insG, followed by FN298674:g.90T>C, FN298674:g.2045C>G, and FN298674:g.4942insT (Table 2). For FN298674:g.1891insG, OR was at 1.71 per allele and at 2.82 among homozygotes and the −log_10_P-values reached a value of 9.3. The ORs for FN298674:g.90T>C were at 0.64 and 0.4, respectively, with −log_10_P-values up to 7.0. For FN298674:g.90T>C, genotype frequencies for all GH cows analysed were at 0.254 (C/C), 0.509 (C/T), and 0.237 (T/T) and for FN298674:g.1891insG at 0.272 (WT/WT), 0.473 (WT/INS) and 0.255 (INS/INS). The overall genotype effect of FN298674:g.90T>C and FN298674:g.1891insG on the LDA frequency showed significant F-values (9.4 and 8.8, respectively) and the contrasts of the least-square means of the LDA frequencies among the homozygous mutant versus the heterozygous genotype or homozygous wildtype were significant. Of all cows carrying the homozygously mutated genotype, 67.3% and 68.1% were affected by LDA. Of the cows homozygous for the wildtype allele, 44.0% and 42.6% were affected by LDA and of the heterozygous individuals, 49.6% and 51.6% were affected (Table 3). Of all 601 LDA-affected animals, only 0.198 and 0.218 were homozygous for the wildtype allele.

**Table 2:** Association study for LDA using SNPs within *motilin* for 1,136 German Holstein cows.

SNP	MAF allele	MAF		OR per allele (95% CI)	OR among homozygotes	PIC	HET	P			
		Cases	Controls					gt	allele	hom	strat
		(n = 601)	(n = 535)		(95% CI)			(−log_10_)	(−log_10_)	(−log_10_)	(−log_10_)
FN298674:g.62G>A	A	0.49	0.39	1.47 (1.23–1.72)	2.27 (3.22–1.59)	0.37	0.47	4.8	5.1	5.4	3.3
FN298674:g.90T>C	T	0.44	0.55	0.64 (0.54–0.76)	0.40 (0.28–0.56)	0.37	0.48	7.0	6.8	6.8	3.8
FN298674:g.1891insG	insG	0.55	0.42	1.71 (1.44–2.02)	2.82 (2.01–3.95)	0.37	0.46	8.2	9.3	9.0	3.7
FN298674:g.2045C>G	G	0.52	0.40	1.62 (1.36–1.93)	2.44 (1.74–3.42)	0.37	0.40	6.6	7.1	6.7	3.2
FN298674:g.4942insT	wt	0.35	0.47	0.61 (0.51–0.72)	0.41 (0.29–0.6)	0.35	0.41	7.0	7.4	5.8	2.9
FN298674:g.6689C>T	T	0.28	0.40	0.57 (0.45–0.72)	0.36 (0.22–0.57)	0.32	0.34	4.3	5.6	5.0	1.6
FN298674:g.6728G>A	A	0.28	0.40	0.57 (0.45–0.72)	0.36 (0.22–0.57)	0.32	0.34	4.3	5.6	5.0	1.6

For each SNP, minor allele frequencies (MAF), the MAF allele, odds ratios (OR) with their 95% confidence intervals (CI), polymorphism information content (PIC), and heterozygosity (HET) are given. Furthermore, −log_10_P-values (P) are given for genotype (gt), allele, among homozygotes (hom) and for genotype after correcting the data for stratification (strat).

**Table 3:** Least-squares means (LSM) with their corresponding standard errors (SE) of the frequency of LDA on the underlying logit scale for the genotypes of the SNPs FN298674:g.90T>C and FN298674:g.1891insG and the contrasts among the LSMs with their test statistics and P-values.

SNP	Genotype or contrast	LDA-affected cows (%)	LSM ± SE or contrast among LSMs	t-value	P-value
FN298674:g.90T>C	T/T	44.0	−0.319±0.176		
	C/T	49.6	−0.287±0.149		
	C/C	67.3	0.393±0.183		
	C/C vs. C/T		0.680±0.166	4.09	<0.0001
	C/C vs. T/T		0.712±0.199	3.57	0.0004
	C/T vs. T/T		0.032±0.167	0.19	0.8
FN298674:g.1891insG	WT/WT	42.6	−0.366±0.169		
	WT/INS	51.6	−0.206±0.149		
	INS/INS	68.1	0.393±0.181		
	WT/WT vs. WT/INS		−0.161±0.162	−1.00	0.3
	WT/WT vs. INS/INS		−0.759±0.193	−3.93	<0.0001
	WT/INS vs. INS/INS		−0.598±0.168	−3.56	0.0004

Genotypic means for the frequency of LDA were estimated through LSMs in a mixed logit model correcting for data stratification.

In order to correct for the data structure, we employed a logit model including a random sire effect besides the fixed genotypic SNP effects. This association analysis showed the most significant −log_10_P-values for FN298674:g.90T>C and FN298674:g.1891insG (3.8 and 3.7). The −log_10_P-values of all other polymorphisms were distinctly smaller. In addition, we performed association analyses using a random additive genetic animal effect through a numerator relationship matrix and a heritability of h^2^ = 0.3 for LDA estimated in the same population [1], [2]. The most significant associations with LDA were found for FN298674:g.90T>C and FN298674:g.1891insG. The FN298674:g.90T>C SNP showed the highest association with LDA. The SNPs FN298674:g.6689C>T and FN298674:g.6728G>A were no longer significantly associated with LDA. Therefore, we considered FN298674:g.90T>C and FN298674:g.1891insG as the most influential SNPs of *MLN* for developing LDA. The phenotypic variance explained after correction for the sire effect was at 3.1% for FN298674:g.90T>C and at 3.9% for both SNPs, FN298674:g.90T>C and FN298674:g.1891insG.

### Haplotype analysis

The most common haplotypes for FN298674:g.90T>C and FN298674:g.1891insG comprising 95% of all cows were C-INS and T-WT. The haplotype including both mutant alleles (C-INS) was present in 55.1% of the LDA-affected and in 39.0% of the unaffected cows (Table S1). The haplotype trait analysis using these SNPs gave a χ^2^ value of 119.4 (p<0.0001) and χ^2^ values of 14.0 (p = 0.0002) to 60.8 (p<0.0001) for the distribution of the four single haplotypes among LDA-affected and control cows. Inclusion of further *MLN* polymorphisms did not improve the haplotype analysis.

### Linkage analysis

We employed a multipoint non-parametric linkage analysis to re-assess linkage for *MLN*-associated markers in the data set previously used for the genome-wide search for LDA-QTL [4]. The chromosome-wide linkage analysis for 30 markers on BTA23 and all 14 paternal half-sib families showed a peak with a Zmean of 2.55 (P-value = 0.005) at the SNP FN298674:g.90T>C within *MLN* (Table S2, Figure S2).

### Distribution of LDA-associated alleles in German Fleckvieh

The most significantly LDA-associated polymorphisms, FN298674:g.90T>C and FN298674:g.1891insG, were tested in 148 German Fleckvieh cattle. German Fleckvieh was used as a reference breed as this breed is known for its very low incidence of LDA. Genotypes of FN298674:g.90T>C were 0.468 T/T, 0.371 C/T and 0.161 C/C. In comparison to GH cows, German Fleckvieh showed a shift to the wildtype allele T and the homozygous wildtype genotype T/T. For FN298674:g.1891insG, the genotypic distribution was 0.591 without insertion (WT/WT), 0.295 heterozygous (WT/INS) and 0.114 homozygously inserted (INS/INS). Allele frequencies for the mutant alleles were 0.347 (FN298674:g.90T>C) and 0.262 (FN298674:g.1891insG).

### Expression study

A qRT-PCR of cDNA gave evidence of a *MLN* expression in abomasal mucosa tissue. To test whether the FN298674:g.90T>C mutation might influence the *MLN* expression, samples of abomasal mucosa tissue from 55 previously genotyped GH cows were taken and analysed using qRT-PCR. Of these individuals, 26 were homozygous for the wildtype allele, 20 were heterozygous, and nine were homozygous for the mutant allele. The mean value of the ΔCT of the homozygous wildtype cows was used as calibrator [11]. The relative expression levels of *MLN* were decreased by 89% in cows being heterozygous or homozygous for the mutant allele C of the polymorphism FN298674:g.90T>C in comparison to cows homozygous for the wildtype allele T (Table 4). Among cows carrying the mutant allele C homozygously or heterozygously, the expression levels were identical. The error probability for the wildtype genotype versus the heterozygous or homozygous mutated genotype was at a P-value of 0.03 with a corresponding F-value of 4.8 (Table 4).

**Table 4:** Results of the expression analysis for FN298674:g.90T>C within *motilin* (*MLN*).

Relative expression levels per genotype		T/T vs. C/T and C/C	
T/T (n = 27)	C/T (n = 20) and C/C (n = 8)	F-value	P-value
1.00±0.26	0.11±0.27	4.8	0.03

Relative expression levels per genotype		C/T vs. C/C	
C/T (n = 20)	C/C (n = 8)	F-value	P-value
0.11±0.31	0.11±0.47	0.2	0.7

The mean relative expression levels of *MLN* with their standard errors are given for the wildtype genotype T/T, the heterozygously (C/T) and the homozygously mutated genotype (C/C) standardized on the mean of the genotype T/T. Furthermore, the F-values and P-values for the differences in mean relative expression levels among the genotype T/T versus the genotypes C/T and C/C as well C/T versus C/C are given. *RPL4* was used as housekeeping gene.

## Discussion

This study demonstrated that the non-coding *MLN* transcription factor binding site mutation FN298674:g.90T>C is significantly associated with *MLN* expression levels and the incidence of LDA in GH cows. Furthermore, this polymorphism showed a significant linkage with LDA in GH cows from the same population. The *MLN* gene encodes the 22 amino-acid peptide motilin, a hormone which plays a role in stimulating gastrointestinal motility. Motilin is considered as an endocrine regulator and initiator of phase III of the migrating motor complex, the interdigestive peristaltic reflex in the gastrointestinal tract [5], [12]. This hormone therefore causes gastric emptying in periods of fasting and its release ceases with the ingestion of food. Plasma levels of motilin increase cyclically every 90–120 minutes within the interdigestive periods, which evoke short time intervals of five to ten minutes of strong peristaltic contractions initiating from the stomach [5]. In a study using the house musk screw as animal model for motilin studies, the contractile responses induced by motilin were dose-dependent [13]. There are also therapeutical approaches in using the positive effect of motilin agonists on gastric contractions concerning the treatment of diabetic gastroparesis [6], [7] and altered motilin plasma levels are seen in some human diseases [14]. This study shows that *MLN* plays a role in the development of bovine LDA, a disease known to be initiated among other things by a reduced peristalsis of the gastrointestinal tract [3]. The reduced food-intake often seen in dairy cows after calving necessitates a high interdigestive peristalsis, which is a function of *MLN*. Naturally, its influence on the development of LDA is a fractional amount, because the disease is known to be genetically complex. After correcting the data for stratification, the polymorphism FN298674:g.90T>C within bovine *MLN* showed the highest association with LDA. This SNP affects a NKX2-5 transcription factor binding site. The effect of NKX2-5 on gene expression has been previously described [15] and SNPs within NKX2-5 binding sites have been reported to be functional for complex genetic diseases as systemic lupus erythematosus, rheumatoid arthritis, and graves disease in man as the mutations lead to a reduced expression of the respective gene [16]. Therefore, FN298674:g.90T>C is presumably the reason for the reduced expression of *MLN* in GH cows carrying a C allele at this position. Interestingly, while the association study showed the highest associations for the homozygous mutant genotype with LDA, the expression of *MLN* was reduced in cows with at least one copy of the mutated allele. This indicates a dominant effect of FN298674:g.90T>C. These results might be explained by the motilin-level regulation process. Motilin controls the gastrointestinal peristalsis reflex to provide gastric emptying during the interdigestive phases [5], [12]. Plasma levels of motilin increase cyclically within these phases and evoke short time intervals of strong peristaltic contractions [5]. Therefore, though cows heterozygous for the FN298674:g.90T>C SNP show a considerably reduced basis expression of *MLN*, they might still be able to raise their motilin level more frequently or higher during the digestive periods where it is required and therefore reach a threshold to prevent LDA. Individuals homozygous for the mutated allele cannot raise their motilin level much beyond basis expression. Therefore, the gastric motility in heterozygous cows might be sufficient to prevent LDA.

Though the homozygously mutated genotype of FN298674:g.90T>C significantly increases the risk of a cow to be affected by LDA and is the most likely causal SNP due to its effect on a transcription factor binding site, FN298674:g.1891insG within the first intron of *MLN* also showed a highly significant association with LDA. As FN298674:g.1891insG is in strong linkage disequilibrium (r^2^ = 0.8) with FN298674:g.90T>C, its high association with LDA might be explained by this fact. However, the insertion mutation FN298674:g.1891insG might also contribute to the negative effect on *MLN* expression since an effect on a further regulatory domain can not be excluded.

To reveal the genotypic distribution of the SNPs FN298674:g.90T>C and FN298674:g.1891insG in a cattle breed with a low incidence of LDA [17], these SNPs were tested in German Fleckvieh. In German Fleckvieh, the wildtype allele was prevalent with a frequency of 0.653 at FN298674:g.90T>C and of 0.738 at FN298674:g.1891insG and the homozygous mutant genotypes for these polymorphisms showed an even lower frequency (0.161 and 0.114, respectively) than in GH cows unaffected by LDA (0.176 and 0.170, respectively). This corroborates the impact of *MLN* as a causal gene for LDA.

GH are high-performance dairy cows and due to their energy adapted-feeding and the cattle specific anatomic peculiarities they might be more susceptible for diseases caused by gastric motility decrease than other species. However, alongside cattle and human [18], gastric dilatation and displacement have also been reported in other species like dogs [19], cats [20], pigs [21], guinea pigs [22], and horses [23]. Therefore, this study might be of interest in the research of gastric motility disorders in these species.

In conclusion, we identified a SNP (FN298674:g.90T>C) which affects a predicted NKX2-5 binding site within the first non-coding exon of the bovine *MLN* gene. This polymorphism was shown to be correlated with a significantly lower expression of *MLN* and a significant increase of LDA incidence in GH cows. This is the first polymorphism showing a functional association with LDA and it can be used to test for LDA-susceptibility in this breed. *MLN* might be a useful candidate for the genetic analysis of gastric motility disorders in further species.

## Materials and Methods

### Ethics statement

All animal work has been conducted according to the national and international guidelines for animal welfare. All blood-sampling of LDA-affected cows was done in university clinics for cattle along with diagnostic protocols prior to surgery. The blood samples of unaffected cows were taken by veterinarians on farms. The project has been notified to the Lower Saxony state veterinary office, Niedersächsisches Landesamt für Verbraucherschutz und Lebensmittelsicherheit, Oldenburg, Germany, and registered as a notifiable experiment with the number 33.9-42502-05-08A541.

### Animals

We used EDTA-blood samples of a total of 1,136 GH cows. Of these animals, 601 cows had undergone a surgery because of LDA at the clinics for cattle of the veterinary universities Hannover or Giessen, Germany. The remaining 535 GH cows were unaffected by LDA. This sample was balanced by paternal grandsires. Each grandsire had to have at least one descendant affected by LDA and one unaffected descendant. The data set included 72 grandsires. Furthermore, EDTA-blood samples of 148 German Fleckvieh individuals with diverse ancestry were included into this study. Genomic DNA from EDTA-blood was extracted using the QIAamp 96 Spin Blood Kit (Qiagen, Hilden, Germany). In addition, samples from the abomasal mucosa tissue were taken from 55 GH cows at the slaughterhouse (Schlachthof Hannover and Schlachthof Giessen, Germany). All these animals were fasting prior to sampling. The samples were taken under equal conditions directly after slaughtering.

### Sequencing and genotyping

The complete *MLN* gene was sequenced and scanned for polymorphisms in each four LDA-affected and unaffected GH cows. To ensure that the four control cows would not develop a LDA later in life, only cows with an age of at least nine years and without ever showing signs of LDA in their lifes were chosen for this first scan for polymorphisms. Sequence data were analysed using the Sequencher 4.9 software (GeneCodes, Ann Arbor, MI). PCR primers used for sequencing of *MLN* and for genotyping are given in Table 1 and Table S3. Sequencing of PCR products was performed on a MegaBACE 1,000 capillary sequencer (GE Healthcare, Freiburg, Germany). After this, each polymorphism identified was tested in 24 LDA-affected cows and 24 controls and, if it was informative, genotyped in a total of 1,136 GH cows. Markers were regarded as informative, if their PIC and heterozygosity were at 0.25 or higher. Genotyping of SNPs was performed using enzymatic digestion or sequencing (Table S4). For the genotyping of microsatellites, insertions, and deletions, each one primer was IRD-labeled and the PCR products were size-fractionated by gel electrophoresis on automated sequencers (LI-COR 4300, Lincoln, NE, USA) using 6% polyacrylamide denaturing gels (Table S4).

### Expression analysis

For the analysis of *MLN* expression, RNA was isolated from abomasal mucosa tissue samples of 55 different cows using the NucleoSpin®RNA II kit (Macherey-Nagel, Düren, Germany) according to the manufacturer's instructions. The reverse transcription into cDNA was performed using 200 U SuperScript III Reverse Transcriptase (Invitrogen, Karlsruhe, Germany). A single PCR assay was used for quantification of the MLN gene transcript (GenBank NM_173938.2) using a forward primer situated on the boundary of exon 2 and exon 3 (5′- TGC AGG AAA AGG AGA GGT ACA AG -3′) and a reverse primer within exon 4 (5′- GTT CAT CCT CAT TCC AAT TTC CA -3′) amplifying an 155-bp product with a FAM-labeled TaqMan minor groove binding (MGB) probe (Applied Biosystems, Darmstadt, Germany) located at the boundary of exon 3 and 4 (5′-CAG GAA GTT ATC AAG CTG A-3′). Bovine *RPL4* was used as a housekeeping gene. We used a forward primer within exon 2 (5′-TTG CGC AAA AAC AAC AGA CA-3′) and a reverse primer in exon 3 (5′-GCC GGT ACC CCA AGA CTC A-3′) amplifying a 81-bp product in combination with a VIC-labeled TaqMan MGB probe (Applied Biosystems) spanning the boundary of exon 2 and 3 (5′-TAG CAG GTC ATC AAA CC-3′) according to the bovine reference mRNA sequence (GenBank NM_001014894). The quantitative real-time (qRT)-PCR was carried out using an ABI7300 sequence detection system (Applied Biosystems). The *MLN* transcript specific expression was normalised by the bovine *RPL4* expression level (ΔCT), and the relative expression level was calculated by the ΔΔCT method using the average ΔCT of the homozygous wildtype samples as calibrator [11]. All assays were performed in duplicates.

### Statistical analyses

To test for association with LDA, case-control analyses based on *χ* ^2^-tests for genotypes and alleles were performed using SAS/Genetics, version 9.3 (Statistical Analysis System, Cary, NC, USA, 2011). The ALLELE and HAPLOTYPE procedures of SAS/Genetics were used for estimation of allele and haplotype frequencies, Hardy-Weinberg equilibrium and linkage disequilibrium among marker alleles. Logistic regression analysis was performed using the GLIMMIX procedure of SAS. The GLIMMIX procedure fits statistical models to data with correlations or nonconstant variability and where the response is not necessarily normally distributed and was therefore also used with our data. An animal model analysis was parameterized using a numerator relationship matrix for each individual and a heritability of 0.3 for LDA. This heritability was chosen as a mean value from former studies for LDA in GH cows where heritabilities of 0.2 to 0.5 were estimated [1], [2]. Odds ratios (OR) and *P*-values were obtained from the CASECONTROL and GLIMMIX procedure. Promoter prediction was carried out by Promoter Prediction software (http://www.fruitfly.org). To detect the impact of intronic polymorphisms and to search for transcription factor binding sites, the TFSEARCH software (http://www.cbrc.jp/research/db/TFSEARCH.html) was used. Statistical calculation of pairwise linkage disequilibrium (LD) was performed using HAPLOVIEW 4.0 (http://www.broad.mit.edu/mpg/haploview/). Statistical evaluation of *MLN* expression results was performed using standard procedures for comparing means with GLM (general linear model) of SAS, version 9.3.

### Linkage Analysis

In addition to the association analysis, a multipoint non-parametric linkage analysis was conducted. For this purpose, 14 previously described paternal half-sib families segregating for LDA and containing a total of 360 animals were employed [4]. These families can be grouped into five grandsire families. We have taken the same microsatellites on BTA23 as in the previous study. In addition, we included the SNPs FN298674:g.90T>C and FN298674:g.1891insG within *MLN* as well as five new microsatellite markers located on the proximal end of BTA23. Positions of all markers were determined using a BLAST (http://blast.ncbi.nlm.nih.gov/Blast.cgi) analysis against the UMD_3.1 bovine genome assembly. The markers *DIK5319* and *BM47* could not be placed or were located on a different chromosome and therefore excluded from this study. A total of 30 markers on BTA23 were used. PCR was conducted according to a standard protocol. One primer of each pair was endlabeled with fluorescent IRD700 or IRD800. Primers and positions of the five newly developed microsatellites are given in Table S5. For the analysis of the microsatellite genotypes, the PCR products were size-fractionated by gel electrophoresis on an automated sequencer (LI-COR 4300, Lincoln, NE, USA) using 6% polyacrylamide denaturing gels. Multipoint non-parametric linkage analyses based on haplotypes identical-by-descent (IBD) for a half-sib design were employed [24], [25]. The statistical analyses were performed using MERLIN, version 1.1.2 [26].

## Supporting Information

Figure S1 **Linkage disequilibrium (LD) among the seven LDA-associated SNPs in German Holstein cows.** The pairwise r^2^-values are shown for each SNP pair. The red square between the markers FN298674:g.6689C>T and FN298674:g.6728G>A indicates complete LD.
(TIF)

Figure S2 **The Zmean score profile with corresponding chromosome-wide P-values for LDA in German Holstein cattle for the bovine chromosome (BTA) 23.** The horizontal line indicates the threshold of chromosome-wide significance (P-value = 0.05).
(TIF)

Table S1 **Haplotype analysis of the polymorphisms FN298674:g.90T>C and FN298674:g.1891insG.** The haplotype frequencies as well as their standard errors, χ^2^-values and P-values are given. The test for marker-trait association gave a χ^2^ value of 119.4 (p<0.0001).
(DOC)

Table S2 **Zmeans and LOD-scores with their P-values of the multipoint non-parametric linkage analysis for BTA23 including the SNPs FN298674:g.90T>C and FN298674:g.1891insG within** ***MLN*** **.** For the analysis, 14 paternal half-sib families comprising 360 individuals were used.
(DOC)

Table S3 **Additional pairs of primers used for sequencing** ***motilin*** **.** For each pair of primers, the amplified region, annealing temperature (AT) and product size (PS) is given.
(DOC)

Table S4 **Methods used for genotyping of informative SNPs.** All SNPs genotyped using RFLPs (restriction fragment length polymorphisms) or IRD (infrared dye) labeled gel electrophoresis are given. Not specified markers were genotyped by sequencing.
(DOC)

Table S5 **Description of the five new microsatellites used for the linkage analysis.** The positions (Pos) determined on the bovine UMD_3.1 assembly, primer sequences, repeat motif, heterozygosity (Het), polymorphism information content (PIC), number of alleles (A), annealing temperature (AT) and product sizes (PS) are given.
(DOC)
