# Supplementary figures and images for: Transcription Factor Binding Site Polymorphism in the Motilin Gene Associated with Left-Sided Displacement of the Abomasum in German Holstein Cattle
Source: PLoS One. 2012 Apr 20;7(4):e35562. doi: 10.1371/journal.pone.0035562 (PMC3334980; doi:10.1371/journal.pone.0035562)

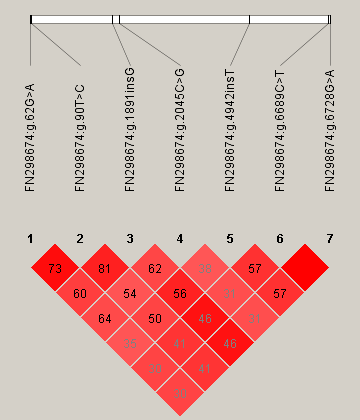

Supplement: Figure S1 — Linkage disequilibrium (LD) among the seven LDA-associated SNPs in German Holstein cows. The pairwise r2-values are shown for each SNP pair. The red square between the markers FN298674:g.6689C>T and FN298674:g.6728G>A indicates complete LD. (TIF) [file pone.0035562.s001.tif]

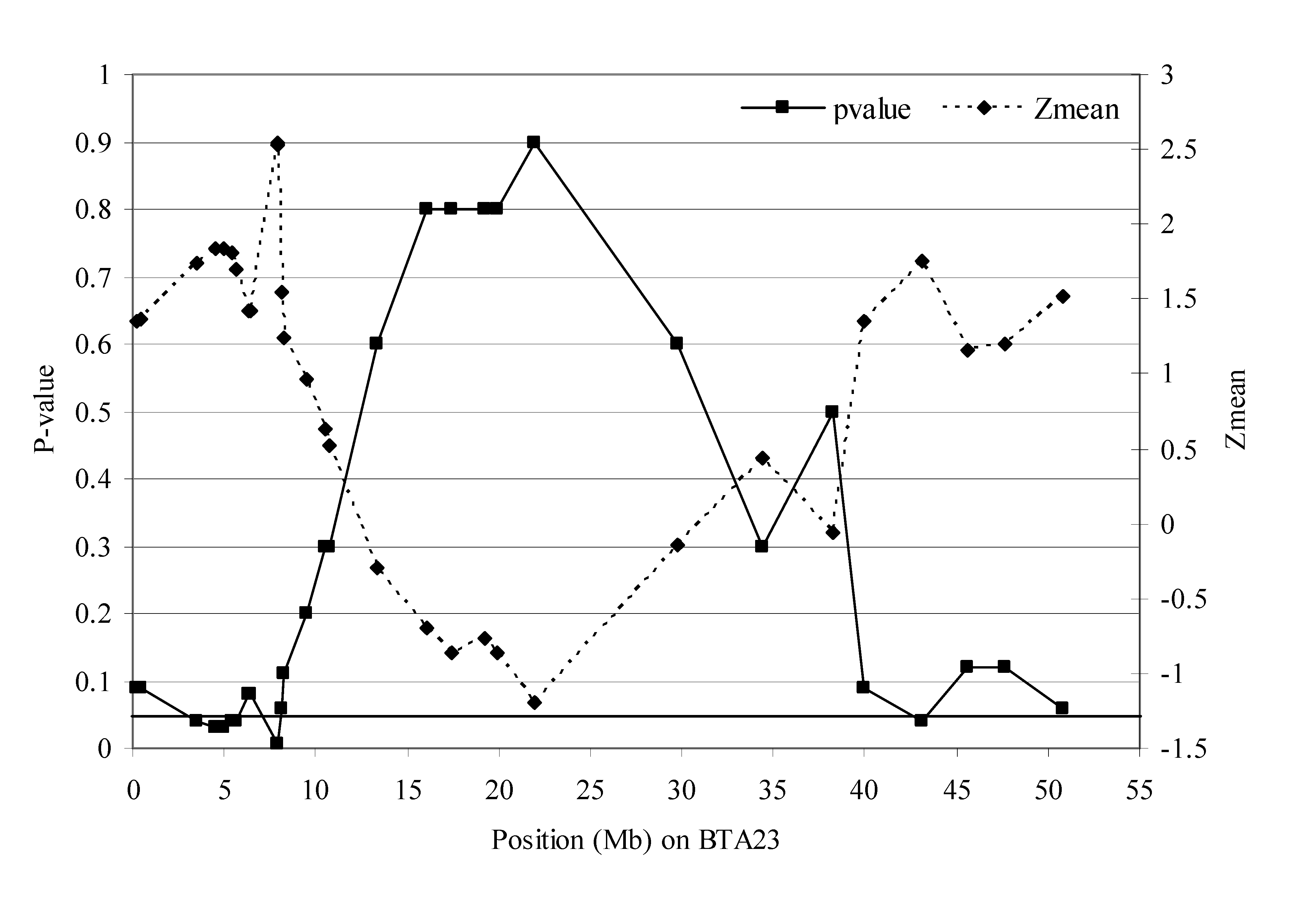

Supplement: Figure S2 — The Zmean score profile with corresponding chromosome-wide P-values for LDA in German Holstein cattle for the bovine chromosome (BTA) 23. The horizontal line indicates the threshold of chromosome-wide significance (P-value = 0.05). (TIF) [file pone.0035562.s002.tif]
