# Supplementary material for: Transcription Factor Binding Site Polymorphism in the Motilin Gene Associated with Left-Sided Displacement of the Abomasum in German Holstein Cattle
Source: PLoS One. 2012 Apr 20;7(4):e35562. doi: 10.1371/journal.pone.0035562 (PMC3334980; doi:10.1371/journal.pone.0035562)
Supplement: Table S1 — Haplotype analysis of the polymorphisms FN298674:g.90T>C and FN298674:g.1891insG. The haplotype frequencies as well as their standard errors, χ2-values and P-values are given. The test for marker-trait association gave a χ2 value of 119.4 (p<0.0001). (DOC) [file pone.0035562.s003.doc]

**Table S1. Haplotype analysis of the polymorphisms FN298674:g.90T>C and FN298674:g.1891insG.** The haplotype frequencies as well as their standard errors, χ^2^-values and *P*-values are given. The test for marker-trait association gave a χ^2^ value of 119.4 (p<0.0001).

| Haplotype | Combined  frequency  (%) | Frequency in  LDA-unaffected  cows (%) | Frequency in  LDA-affected  cows (%) | Standard error  (%) | χ^2^ | *P* value |
| --- | --- | --- | --- | --- | --- | --- |
| C – wt | 3.3 | 6.2 | 0.9 | 0.4 | 49.2 | <0.0001 |
| C – insG | 47.5 | 38.6 | 55.1 | 1.1 | 60.8 | <0.0001 |
| T – wt | 47.5 | 51.6 | 43.7 | 1.1 | 14.0 | 0.0002 |
| T – insG | 1.7 | 3.6 | 0.3 | 0.3 | 35.2 | <0.0001 |

wt: wildtype

insG: insertion of a G
