# Supplementary material for: Transcription Factor Binding Site Polymorphism in the Motilin Gene Associated with Left-Sided Displacement of the Abomasum in German Holstein Cattle
Source: PLoS One. 2012 Apr 20;7(4):e35562. doi: 10.1371/journal.pone.0035562 (PMC3334980; doi:10.1371/journal.pone.0035562)
Supplement: Table S2 — Zmeans and LOD-scores with their P-values of the multipoint non-parametric linkage analysis for BTA23 including the SNPs FN298674:g.90T>C and FN298674:g.1891insG within MLN . For the analysis, 14 paternal half-sib families comprising 360 individuals were used. (DOC) [file pone.0035562.s004.doc]

**Table S2. Zmeans and LOD-scores with their P-values of the multipoint non-parametric linkage analysis for BTA23 including the SNPs FN298674:g.90T>C and FN298674:g.1891insG within *MLN*. For the analysis, 14 paternal half-sib families comprising 360 individuals were used.**

| Marker | Position  (Mb) | Zmean | p-value | LOD | p-value |
| --- | --- | --- | --- | --- | --- |
| *MS_KHDRBS2* | 0.2 | 1.35 | 0.09 | 0.52 | 0.06 |
| *NLBCMK15* | 0.5 | 1.37 | 0.09 | 0.52 | 0.06 |
| *DIK5099* | 3.5 | 1.74 | 0.04 | 0.69 | 0.04 |
| *INRA064* | 4.5 | 1.83 | 0.03 | 0.71 | 0.04 |
| *IOBT528* | 5.0 | 1.83 | 0.03 | 0.7 | 0.04 |
| *MS_FAM83B* | 5.4 | 1.81 | 0.04 | 0.68 | 0.04 |
| *DIK4865* | 5.7 | 1.7 | 0.04 | 0.61 | 0.05 |
| *SRC119* | 6.4 | 1.42 | 0.08 | 0.46 | 0.07 |
| *CSSM5* | 6.4 | 1.42 | 0.08 | 0.46 | 0.07 |
| **FN298674:g.90T>C** | **7.9** | **2.55** | **0.005** | **0.81** | **0.03** |
| **FN298674:g.1891insG** | **7.9** | **2.53** | **0.006** | **0.8** | **0.03** |
| *DIK2097* | 8.1 | 1.54 | 0.06 | 0.5 | 0.07 |
| *MS_NUDT3* | 8.3 | 1.24 | 0.11 | 0.36 | 0.1 |
| *MS_LDA_9,5* | 9.5 | 0.97 | 0.2 | 0.23 | 0.15 |
| *MS_LDA_10,5* | 10.5 | 0.63 | 0.3 | 0.1 | 0.3 |
| *DIK4895* | 10.8 | 0.52 | 0.3 | 0.06 | 0.3 |
| *RM033* | 13.4 | -0.3 | 0.6 | -0.02 | 0.6 |
| *BM1815* | 16.1 | -0.7 | 0.8 | -0.08 | 0.7 |
| *UWCA1* | 17.4 | -0.87 | 0.8 | -0.11 | 0.8 |
| *BM1258* | 19.2 | -0.77 | 0.8 | -0.09 | 0.7 |
| *NRKM030* | 19.9 | -0.87 | 0.8 | -0.11 | 0.8 |
| *DIK5016* | 21.9 | -1.2 | 0.9 | -0.16 | 0.8 |
| *BMS2275* | 29.8 | -0.15 | 0.6 | -0.01 | 0.6 |
| *RM185* | 34.4 | 0.44 | 0.3 | 0.03 | 0.4 |
| *BM7233* | 38.3 | -0.06 | 0.5 | 0 | 0.5 |
| *DIK2608* | 39.9 | 1.35 | 0.09 | 0.37 | 0.1 |
| *CSSM24* | 43.1 | 1.76 | 0.04 | 0.7 | 0.04 |
| *BM1905* | 45.6 | 1.16 | 0.12 | 0.33 | 0.11 |
| *DIK4203* | 47.7 | 1.2 | 0.12 | 0.33 | 0.11 |
| *DIK2066* | 50.8 | 1.52 | 0.06 | 0.48 | 0.07 |
