# Supplementary material for: Transcription Factor Binding Site Polymorphism in the Motilin Gene Associated with Left-Sided Displacement of the Abomasum in German Holstein Cattle
Source: PLoS One. 2012 Apr 20;7(4):e35562. doi: 10.1371/journal.pone.0035562 (PMC3334980; doi:10.1371/journal.pone.0035562)
Supplement: Table S3 — Additional pairs of primers used for sequencing motilin . For each pair of primers, the amplified region, annealing temperature (AT) and product size (PS) is given. (DOC) [file pone.0035562.s005.doc]

**Table S3.** **Additional pairs of primers used for sequencing *MLN.*** For each pair of primers, the amplified region, annealing temperature (AT) and product size (PS) is given.

| Primer F (5’ > 3’) | Primer R (5’ > 3’) | Amplified  region | AT | PS |
| --- | --- | --- | --- | --- |
| ACGAATCCTGGCAAAAGAGA | CTGCTCACTTTCGTGATCCA | intron 1 | 60 | 453 |
| TCAGATGACCGAGGAGACG | TCCAGAAACAACCTCCTTCC | intron 1 | 60 | 736 |
| CGGGGGAAGTTTCCAATTA | AAAGGTACTCAGAACTCCATGACA | intron 2 | 60 | 515 |
| CCCTGGGCCTTTTACCTTAG | TTGTGTATGGGTCCCTGTTG | intron 2 | 60 | 627 |
| GTGCTACACGAACTGGCAAA | GCCCCTTGTACCTCTCCTTT | intron 2, exon 3 | 60 | 591 |
| TCCAAGCTGGGATGGGTA | TTCCCCTTCCCCTGAATTAT | intron 3 | 60 | 652 |
| TGCAAACCTCCTTTGCTTTT | GCGTGTAGCCACACAGTGAT | intron 4, exon 5, 3’UTR | 60 | 671 |
| GGACACCAGCAAAAAGCAA | GTTTTTCTGCTTCTCGGAGTG | 3’UTR | 60 | 647 |
