# Supplementary material for: Transcription Factor Binding Site Polymorphism in the Motilin Gene Associated with Left-Sided Displacement of the Abomasum in German Holstein Cattle
Source: PLoS One. 2012 Apr 20;7(4):e35562. doi: 10.1371/journal.pone.0035562 (PMC3334980; doi:10.1371/journal.pone.0035562)
Supplement: Table S4 — Methods used for genotyping of informative SNPs. All SNPs genotyped using RFLPs (restriction fragment length polymorphisms) or IRD (infrared dye) labeled gel electrophoresis are given. Not specified markers were genotyped by sequencing. (DOC) [file pone.0035562.s006.doc]

**Table S4.** **Methods used for genotyping of informative SNPs.** All SNPs genotyped using RFLPs (restriction fragment length polymorphisms) or IRD (infrared dye) labeled gel electrophoresis are given. Not specified markers were genotyped by sequencing.

| Amplified markers within *MLN* | Method of genotyping | Restriction enzyme  or IRD labeling |
| --- | --- | --- |
| FN298674:g.62G>A | RFLP | HaeIII |
| FN298674:g.90T>C | RFLP | XcmI |
| FN298674:g.2045C>G | RFLP | BsiEI |
| FN298674:g.6689C>T | RFLP | CviQI |
| FN298674:g.6728G>A | RFLP | HhaI |
| FN298674:g.551(AG)45 | Product size determination  (LI-COR 4300) | IRD800 labeling |
| FN298674:g.1891insG | Product size determination  (LI-COR 4300) | IRD800 labeling |
| FN298674:g.4942insT | Product size determination  (LI-COR 4300) | IRD700 labeling |
