# Supplementary material for: Transcription Factor Binding Site Polymorphism in the Motilin Gene Associated with Left-Sided Displacement of the Abomasum in German Holstein Cattle
Source: PLoS One. 2012 Apr 20;7(4):e35562. doi: 10.1371/journal.pone.0035562 (PMC3334980; doi:10.1371/journal.pone.0035562)
Supplement: Table S5 — Description of the five new microsatellites used for the linkage analysis. The positions (Pos) determined on the bovine UMD_3.1 assembly, primer sequences, repeat motif, heterozygosity (Het), polymorphism information content (PIC), number of alleles (A), annealing temperature (AT) and product sizes (PS) are given. (DOC) [file pone.0035562.s007.doc]

**Table S5. Description of the five new microsatellite markers used for the linkage analysis.** The positions (Pos) determined on the bovine UMD_3.1 assembly, primer sequences, repeat motif, heterozygosity (Het), polymorphism information content (PIC), number of alleles (A), annealing temperature (AT) and product sizes (PS) are given.

| Microsatellite  marker | Pos  (Mb) | Primer F (5’ > 3’) | Primer R (5’ > 3’) | Motif | Het | PIC | A | AT | PS  (bp) |
| --- | --- | --- | --- | --- | --- | --- | --- | --- | --- |
| *MS_KHDRBS2* | 0.2 | tttgacaaattgtggtttcct | gggttgcaaacaagaagcta | (TG)_24_ | 0.64 | 0.56 | 8 | 58 | 181 |
| *MS_FAM83B* | 5.4 | agcacagtcaattcagcctc | aggaaaaggcacctgtcaat | (GT)_25_ | 0.42 | 0.41 | 3 | 58 | 142 |
| *MS_NUDT3* | 8.3 | attttgtgggtgggaaatct | caggctcaacacttcctttcta | (CA)_22_ | 0.69 | 0.62 | 8 | 58 | 184 |
| *MS_LDA_9,5* | 9.5 | agctgaaggaagaaagaacaca | ttacggcagcactaggaaacta | (AC)_19_ | 0.58 | 0.51 | 5 | 58 | 187 |
| *MS_LDA_10,5* | 10.5 | gagtcgttctcaaccccac | gtctggtgtagtgtgcaaggta | (CA)_17_ | 0.64 | 0.52 | 6 | 58 | 116 |
